# Neuroprotective effects of *Gastrodia elata* Blume on promoting M2 microglial polarization by inhibiting JNK/TLR4/T3JAM/NF-κB signaling after transient ischemic stroke in rats

**DOI:** 10.3389/fphar.2024.1469602

**Published:** 2024-09-25

**Authors:** Shang-Chih Huang, Hui-Chi Huang, Wen-Ling Liao, Shung-Te Kao, Chin-Yi Cheng

**Affiliations:** ^1^ Department of Neurology, China Medical University Hospital, Taichung, Taiwan; ^2^ School of Chinese Medicine, College of Chinese Medicine, China Medical University, Taichung, Taiwan; ^3^ Graduate Institute of Integrated Medicine, College of Chinese Medicine, China Medical University, Taichung, Taiwan; ^4^ School of Post-Baccalaureate Chinese Medicine, College of Chinese Medicine, China Medical University, Taichung, Taiwan; ^5^ Department of Chinese Medicine, Hui-Sheng Hospital, Taichung, Taiwan

**Keywords:** *Gastrodia elata* Blume, cerebral ischemia, C-Jun N-terminal kinase, toll-like receptor 4, TRAF3-interacting JNK-activating modulator

## Abstract

**Background:**

*Gastrodia elata* Blume, also called Tian Ma (TM), has been used to treat stroke for centuries. However, its effects on inflammation in acute cerebral ischemic injury and underlying mechanisms involved in microglial polarization remain unknown. The present study explored the effects of the TM extract on the modulation of microglial M1/M2 polarization 2 days after transient cerebral ischemia.

**Methods:**

Male Sprague Dawley rats were intracerebroventricularly administered with 1% dimethyl sulfoxide 25 min before cerebral ischemia and subsequently intraperitoneally administered 0.25 g/kg (DO + TM-0.25 g), 0.5 g/kg (DO + TM-0.5 g), or 1 g/kg (DO + TM-1 g) of the TM extract after cerebral ischemia onset.

**Results:**

DO + TM-0.5 g and DO + TM-1 g treatments downregulated the following: phospho-c-Jun N-terminal kinase (p-JNK)/JNK, tumor necrosis factor (TNF) receptor-associated factor 3 (TRAF3), TRAF3-interacting JNK-activating modulator (T3JAM), p-nuclear factor-kappa B p65 (p-NF-κB p65)/NF-κB p65, ionized calcium-binding adapter molecule 1 (Iba1), CD86, TNF-α, interleukin (IL)-1β, and IL-6 expression and toll-like receptor 4 (TLR4)/Iba1, CD86/Iba1, and p-NF-κB p65/Iba1 coexpression. These treatments also upregulated IL-10, nerve growth factor, and vascular endothelial growth factor A expression and YM-1/2/Iba1 and IL-10/neuronal nuclei coexpression in the cortical ischemic rim. The JNK inhibitor SP600125 exerted similar treatment effects as the DO + TM-0.5 g and DO + TM-1 g treatments.

**Conclusion:**

DO + TM-0.5 g and DO + TM-1 g/kg treatments attenuate cerebral infarction by inhibiting JNK-mediated signaling. TM likely exerts the neuroprotective effects of promoting M1 to M2 microglial polarization by inhibiting JNK/TLR4/T3JAM/NF-κB-mediated signaling in the cortical ischemic rim 2 days after transient cerebral ischemia.

## Introduction

In acute cerebral ischemia, the major pathological mechanism underlying neurological injury is the inflammatory response and the involvement of resident microglia (Xiang et al., 2018; Song et al., 2021). The neuroinflammation and immune reactions that occur after cerebral ischemia/reperfusion (I/R) injury can exacerbate cerebral infarction (Gan et al., 2020). Toll-like receptors (TLRs), one of the transmembrane pattern recognition receptor families, expressed on antigen-presenting cells, including microglia and macrophages, can respond to damage-associated molecular patterns (DAMPs), inducing inflammatory reaction (Ashayeri et al., 2021). TLR4 triggers downstream inflammatory cascades through myeloid differentiation primary response protein 88 (MyD88)/tumor necrosis factor (TNF) receptor-associated factor (TRAF) 6- and TRAF3/TRAF3-interacting c-Jun N-terminal kinase (JNK)-activating modulator (T3JAM)-mediated nuclear factor-kappa B (NF-κB) activation. Studies reported that the TRAF3/T3JAM signaling pathway is closely related to the activation of JNK- and TLR4/NF-κB-mediated signaling (Dadgostar et al., 2003; Li et al., 2019). Furthermore, TLR4-mediated signaling pathways activate the downstream targets JNK and p38 mitogen-activated protein kinase (MAPK), which in turn induce TLR4 upregulation, amplifying TLR4/NF-κB-mediated signaling and exacerbating infarct expansion in the acute stage of ischemic stroke (Cheng et al., 2021; Dong et al., 2021). Thus, the interaction between JNK (p38 MAPK) and TLR4 plays a crucial role in TLR4/T3JAM/NF-κB-associated inflammatory cascades (Cheng et al., 2021). NF-κB, a proinflammatory transcription factor, is expressed in glial cells as a heterodimer of RelA (p65) and p50 subunits; it binds to its endogenous inhibitor IκB in the inactive state. The interaction of TLR4 with DAMPs triggers the activation of IκB kinase, phosphorylating and degrading IκB and resulting in the nuclear translocation of NF-κB and the production of inflammation-related genes in cerebral ischemia (Jover-Mengual et al., 2021). Moreover, the phosphorylation of NF-κB p65 (S536) results in its decreased affinity for IκBα, causing excessive activation and nuclear translocation of NF-κB p65 (Christian et al., 2016). In response to cerebral ischemia, the cytosolic multiprotein complexes of inflammasomes are formed, leading to the secretion of proinflammatory mediators (de Zoete et al., 2014). Among inflammasomes, nucleotide-binding oligomerization domain and leucine-rich repeat (NLR) family pyrin domain containing 3 (NLRP3) is one of the best characterized components of the NLR family that mediates caspase-1 activation and subsequently induces the secretion of proinflammatory factors, such as interleukin-1β (IL-1β) and IL-18, thereby initiating inflammatory cascades (Kelley et al., 2019). By contrast, inhibition of inflammasome components, such as NLRP3 and caspase-1, can alleviate cerebral infarct expansion in acute ischemic stroke (Luo et al., 2022).

The TLR4/JNK (p38 MAPK)/NF-κB-mediated inflammatory signaling pathway is positively correlated with glial NLRP3 inflammasome activity and plays a vital role in microglial activation and polarization (Dong et al., 2021; Jover-Mengual et al., 2021). Microglia are involved in both central nervous system injury and recovery, and these functions are linked to their ability for polarization into the proinflammatory (M1) or anti-inflammatory (M2) phenotype after activation (Ye et al., 2019). In the early stage of cerebral ischemic insults, TLR4-induced microglia are rapidly activated and polarized into M1 and M2 phenotypes in response to the inflammatory cascade and M1 and M2 microglia are characterized by the markers CD86 and YM-1/2, respectively (Wen et al., 2018; Wang et al., 2020; Dong et al., 2021). Within 24 h after cerebral ischemia onset, activated microglia that migrate or infiltrate into the ischemic area are mainly polarized into the M2 phenotype, and they release neurotrophic factors and anti-inflammatory mediators, promoting myelin regeneration, debris clearance, and nerve repair in the ischemic core. Subsequently, the activated microglia are polarized into the proinflammatory M1 phenotype, initiating the release of proinflammatory factors in the peri-infarct area, thereby aggravating ischemic stroke (Xiang et al., 2018; Yusuying et al., 2022). Thus, the increased M1 to M2 ratio is closely related to ischemic injury during transient focal cerebral ischemia, whereas pharmacological interventions for promoting M2 microglia protect against ischemic brain damage (Dong et al., 2021; Wang et al., 2022).

*Gastrodia elata* Blume (also called Tian Ma [TM]), a well-known traditional Chinese herbal medicine, has long been used for treating convulsions, dizziness, headache, limb numbness, and stroke (Wang et al., 2019). TM reduces cerebral infarct partly by inhibiting TNF-α expression in the cortical ischemic rim in acute ischemic stroke (Seok et al., 2019). Gastrodin and parishin C are the biologically active compounds isolated from TM (Lin et al., 2021). Gastrodin ameliorates ischemic stroke by downregulating TNF-α and IL-1β expression in the ischemic area in the acute (Peng et al., 2015) and subacute (Liu et al., 2016) stages of transient middle cerebral artery (MCA) occlusion. Its anti-inflammatory effects are partly due to the activation of nuclear factor erythroid 2-related factor 2-mediated signaling in the ischemic region after transient MCA occlusion (Peng et al., 2015). Parishin C exerts neuroprotective effects partly by suppressing TNF-α, IL-1β, and IL-6 expression in the cortical ischemic rim in the acute stage of transient MCA occlusion (Wang et al., 2021). Anisalcohol derived from TM has been reported to exert anti-neuroinflammatory effects by inhibiting M1 polarization and promoting M2 polarization in lipopolysaccharide-stimulated BV2 microglial cells (Xiang et al., 2018). Taken together, these findings indicate that TM and its main ingredients might provide beneficial effects against ischemic stroke by inhibiting proinflammatory factor expression during cerebral ischemia. However, the detailed pathway involved in the modulation of microglial polarization of the TM extract in acute ischemic stroke remains unclear. In this study, we explored the anti-inflammatory effects of the TM extract on microglia M1 and M2 polarization in the cortical ischemic rim 2 days after transient MCA occlusion.

## Materials and methods

### TM extract preparation

TM extract powder (Batch Number A0418102) was obtained from Kaohsiung Chuang Song Zong (KCSZ) Pharmaceutical Co., Ltd. (Taiwan). The dried rhizomes of TM were imported from Sichuan Province, China, and their quality was evaluated in KCSZ Ligang Laboratory (Taiwan). The data and voucher specimens from KCSZ Ligang Laboratory were confirmed by Professor Jin-Pin Lin (China Medical University, Taiwan) based on the Chinese Pharmacopoeia (11th Edition). TM extract powder was produced as follows: TM dry rhizomes were boiled in boiling water for 1.5 h, and the aqueous extract of TM was obtained. The aqueous extract and corn starch as an excipient were granulated and dried in a fluidized bed dryer. Each gram of the TM extract powder comprised 0.5 g each of the TM extract and corn starch. The TM extract solution was made by dissolving 2 g TM extract powder in 8 mL of normal saline and then centrifuging at 1,000 g at 4°C for 10 min. The supernatant fraction of the TM extract solution (0.125 g/mL) was used for TM treatment.

### Ultra-performance liquid chromatography analysis of the TM extract marker

Gastrodin (>98% purity; Sunhank Technology, Tainan, Taiwan) was dissolved in methanol (10 mg/mL) and used to make six standard solutions (concentration ranging from 1.0 to 0.01562 mg/mL) for ultra-performance liquid chromatography (UPLC). The sample solution of the TM extract was prepared by dissolving it in methanol. A Shimadzu LC-2060C 3D UPLC system (Kyoto, Japan) equipped with a Fortis C18 column (250 × 4.6 mm, 5 μm; maintained at 30°C) was used to screen and identify the chemical marker. Next, 5 μL of a gastrodin standard or TM extract solution was injected using a mobile phase of water (solvent A) and methanol (solvent B) (v/v). The UPLC was programmed as follows: 0–10 min, 10% B; 10–30 min, 10% B to 70% B. The flow rate (1.0 mL/min) and the photodiode array detector (220 nm) were set.

### Animals and transient MCA occlusion

Male Sprague Dawley rats (300–320 g) were purchased from Bio-LASCO (Taipei, Taiwan). The rats used in this study were housed under standard laboratory conditions (room temperature [RT]: 22°C ± 2°C; relative humidity: 55% ± 10%; light/dark cycle: 12/12 h) with free access to water and food. The sample size used in this study was determined based on our previous studies (Cheng et al., 2021; Tsai et al., 2022) and that could offer sufficient statistical power. All the animal experiments were conducted strictly in line with the guidelines of the Institutional Animal Care and Use Committee of China Medical University (No. CMUIACUC-2021-318). The rat model of MCA occlusion was established as previously described (Tsai et al., 2022). In brief, the rats were initially anesthetized with 5% isoflurane and fixed to the stereotaxic frame by the ear bars. Their skulls were exposed by cutting the scalp skins transversely. A small hole was made in the skull (right side, 1.5 mm posterior to the bregma and lateral to the midline). A nylon monofilament (0.2 mm in diameter) was introduced into the internal carotid artery for interrupting the blood flow to the MCA, causing MCA occlusion. After 2 h of MCA occlusion, the nylon monofilament was carefully withdrawn to allow MCA reperfusion. The establishment of the MCA occlusion model was verified through changes in the MCA blood flow, which were monitored using a laser Doppler blood flowmeter (DRT4; Moor Instruments, Wilmington, DE, United States). Successful establishment of the MCA occlusion model was defined as a reduction in the MCA blood flow to 10%–20% of baseline during the ischemia period and an increase in the MCA blood flow to 70% of baseline during the reperfusion period. In the ischemia period, the rats were awake and subjected to modified neurological severity score (mNSS) tests to assess neurological function. Except in the DO + Sham group, the rats with neurological function scores (NFSs) less than 7 revealed incomplete MCA occlusion and were excluded from the study. All the experimental animals were administered intramuscularly with ketorolac, a non-steroidal anti-inflammatory drug, at 1 mg/kg after surgery to reduce pain, suffering and distress.

### Neurological evaluation

The rats were subjected to mNSS tests to evaluate neurological function at 1 and 2 days after reperfusion for motor (muscle strength and walking ability), sensory (placing and proprioception), balance, and reflex (pinna reflex, corneal reflex, and startle reflex) testing, as previously described (Cheng et al., 2021). NFSs were determined based on the neurological examination results, which range from 0 (normal) to 18 (maximal neurological deficit). In this study, the neurological evaluation and cell identification and counting were performed by an experienced laboratory assistant who was blinded to the grouping state.

#### Experiment A

##### Grouping

Thirty-six rats were randomly assigned to six groups (n = 6 each): DO + Sham, DO + Saline, DO + TM-0.25 g, DO + TM-0.5 g, DO + TM-1 g, and SP groups. The doses of the TM extract administered in the TM treatment groups were determined through preliminary experiments. The TM treatment groups were administered intracerebroventricularly with 1% dimethyl sulfoxide (DMSO) 25 min before MCA occlusion; after MCA occlusion onset, the rats were injected intraperitoneally with the TM extract at 0.25 g/kg for the DO + TM-0.25 g group, 0.5 g/kg for the DO + TM-0.5 g group, and 1 g/kg for the DO + TM-1 g group. After 2 days of reperfusion, the rats were subjected to neurological evaluation; they were then killed through CO_2_ inhalation (flow rate: 5.5 L/min), and their brains were harvested. The DO + Saline group was treated in the same protocols as the DO + TM-1 g group, except normal saline was administered instead of the TM extract. The DO + Sham group was treated in the same protocols as the DO + Saline group; however, the MCA was not blocked. The SP group was treated in the same protocols as the DO + Saline group; however, the rats were administered intracerebroventricularly with SP600125, a JNK inhibitor, instead of 1% DMSO.

### Intracerebroventricular administration of 1% DMSO or SP600125

Twenty-five minutes before MCA occlusion, the rats were administered intracerebroventricularly with 10 μL of 1% DMSO or 10 μL of SP600125 solution (2 mM in 1% DMSO, ab120065, Abcam, Waltham, MA, United States) through a burr hole in the right side of the skull using a microsyringe (10 μL, Hamilton Company, Reno, NV, United States), as previously described (Cheng et al., 2021).

### Cerebral infarction assessment

The harvested rat brains were cut into six 2-mm-thick sections for cerebral infarct assessment, as previously described (Cheng et al., 2021). In brief, the sections were stained using 2% 2,3,5-triphenyltetrazolium chloride (TTC; Merck, St. Louis, MO, United States) at 37°C for 5 min. Quantification of the percentage of cerebral infarct areas (divided by the total coronal sectional area) was performed using ImageJ (NIH, Bethesda, MD, United States).

#### Experiment B

##### Grouping

Thirty rats were randomly assigned to six groups (n = 5 each)—DO + Sham, DO + Saline, DO + TM-0.25 g, DO + TM-0.5 g, DO + TM-1 g, and SP groups—with the same procedures and protocols as those described for Experiment A.

### Western blot analysis

In the harvested rat brains, The MCA territory was separated and removed. The right cortical ischemic rims (between 3 and 9 mm from the ischemic core) that were cut from the separated ischemic cortices were collected for Western blot analysis, as previously described (Cheng et al., 2017). The homogenized protein samples were separated based on size through sodium dodecyl sulfate–polyacrylamide gel (10%) electrophoresis. After electrophoresis, the separated proteins were transferred from the gel onto a nitrocellulose (NC) membrane (Hybond-c Extra, Amersham Biosciences, United Kingdom) in transfer buffer. The NC membrane was incubated in 5% skim milk containing 0.1% Tween 20 at RT for 60 min to block nonspecific binding. The target proteins on NC membranes were incubated with primary antibodies diluted in phosphate buffered saline (PBS) containing 5% bovine serum albumin (BSA) (Table 1) at 4°C overnight and then with goat anti-rabbit (1:5000 in PBS/5% BSA, AB_2313567, Jackson, PA, United States) or goat anti-mouse (1:5000 in PBS/5% BSA, AB_10015289, Jackson, PA, United States) IgG secondary antibody at RT for 1 h. Finally, the NC membranes were incubated with an enhanced chemiluminescence reagent (ECL-plus GE Healthcare) and then detected using the FUJIFILM luminescent image analyzer (LAS-3000, Tokyo, Japan). Densitometric analysis of Western blots was performed using ImageJ software.

**TABLE 1:** Primary antibodies used in the present study.

Host	Primary antibody	Western blot (Dilution)	IF (Dilution)	Supplier/catalog No.
Rab	p-JNK	1:1000		CST/^#^9251
Rab	JNK	1:1000		CST/^#^9252
Rab	p-p38 MAPK	1:1000		CST/^#^9211
Rab	p38 MAPK	1:1000		CST/^#^9212
Rab	TRAF6	1:1000		abcam/ab40675
Rab	TRAF3	1:500		abcam/ab36988
Rab	T3JAM	1:500		Merck/SAB4503206
Rab	p-NF-κB p65	1:1000	1:200	CST/^#^3033
Rab	NF-κB p65	1:1000		abcam/ab16502
Rab	NLRP3	1:1000		abcam/ab263899
Rab	Iba1	1:1000	1:100	abcam/ab178846
Rab	Nrf2	1:1000		abcam/ab92946
Rab	VEGF-A	1:1000		Proteintech/^#^19003-1-AP
Mou	CD86	1:1000	1:100	abcam/ab220188
Rab	TNF-α	1:1000		abcam/ab205587
Mou	IL-6	1:2000		abcam/ab9324
Rab	YM-1/2	1:10000	1:100	abcam/ab192029
Rab	IL-10	1:1000		abcam/ab192271
Rab	NGF	1:1000		abcam/ab6199
Mou	Actin (loading control)	1:5000		Novus Biologicals/NB600-501
Mou	TLR4		1:100	abcam/ab22048
Mou	Iba1		1:100	abcam/ab283319
Rab	Il-10		1:100	abcam/ab9969
Mou	NeuN		1:100	Merck Millipore/MAB377

Rab, Rabbit; Mou, Mouse; CST, cell signaling technology; VEGF-A, vascular endothelial growth factor A; NGF, nerve growth factor; NeuN, neuronal nuclei.

#### Experiment C

##### Grouping

Thirty rats were randomly assigned to six groups (n = 5 each)—DO + Sham, DO + Saline, DO + TM-0.25 g, DO + TM-0.5 g, DO + TM-1 g, and SP groups—with the same procedures and protocols as those described for Experiment A.

### Double immunofluorescence staining

The harvested rat brains were embedded in tissue freezing medium, frozen, and cut into 15-µm thick slices, which were used for double immunofluorescence (IF) staining, as previously described (Cheng et al., 2020). The brain slices were postfixed with 4% paraformaldehyde at RT for 15 min, stained with primary antibodies diluted in PBS/5% BSA (revealed in Table 1) at 4°C overnight, and simultaneously stained with DyLight 594-conjugated AffiniPure goat anti-mouse (red, 1:100 in PBS/5% BSA, AB_2338871, Jackson, PA, United States) and DyLight 488-conjugated AffiniPure goat anti-rabbit (green, 1:100 in PBS/5% BSA, AB_2338046, Jackson, PA, United States) IgG secondary antibodies at 37°C for 1.5 h. The colocalization of IF signals of two markers more than or equal to 50% in a cell was defined as double-labeled positive. Immunopositive cells in the selected cortical ischemic rim were counted in nine fields of view at 400 × magnification using fluorescence microscopic methods (CKX53; Olympus, Tokyo, Japan). The double-labeled TLR4/Iba1, CD86/Iba1, p-NF-κB p65/Iba1, YM-1/2/Iba1, and IL-10/NeuN cells were counted and expressed as percentage of immunopositive cells in the selected cortical ischemic rim.

### Statistical analysis

All the experimental data, except the neurological examination, were investigated using one-way ANOVA with *post hoc* Bonferroni test and are presented as mean ± standard deviation. The data obtained from the neurological examination were investigated using one-way ANOVA with Mann-Whitney U test and are presented as median (range). In these tests, p values <0.05 were deemed statistically significant.

## Results

### UPLC analysis of the marker of the TM extract

**FIGURE 1:**
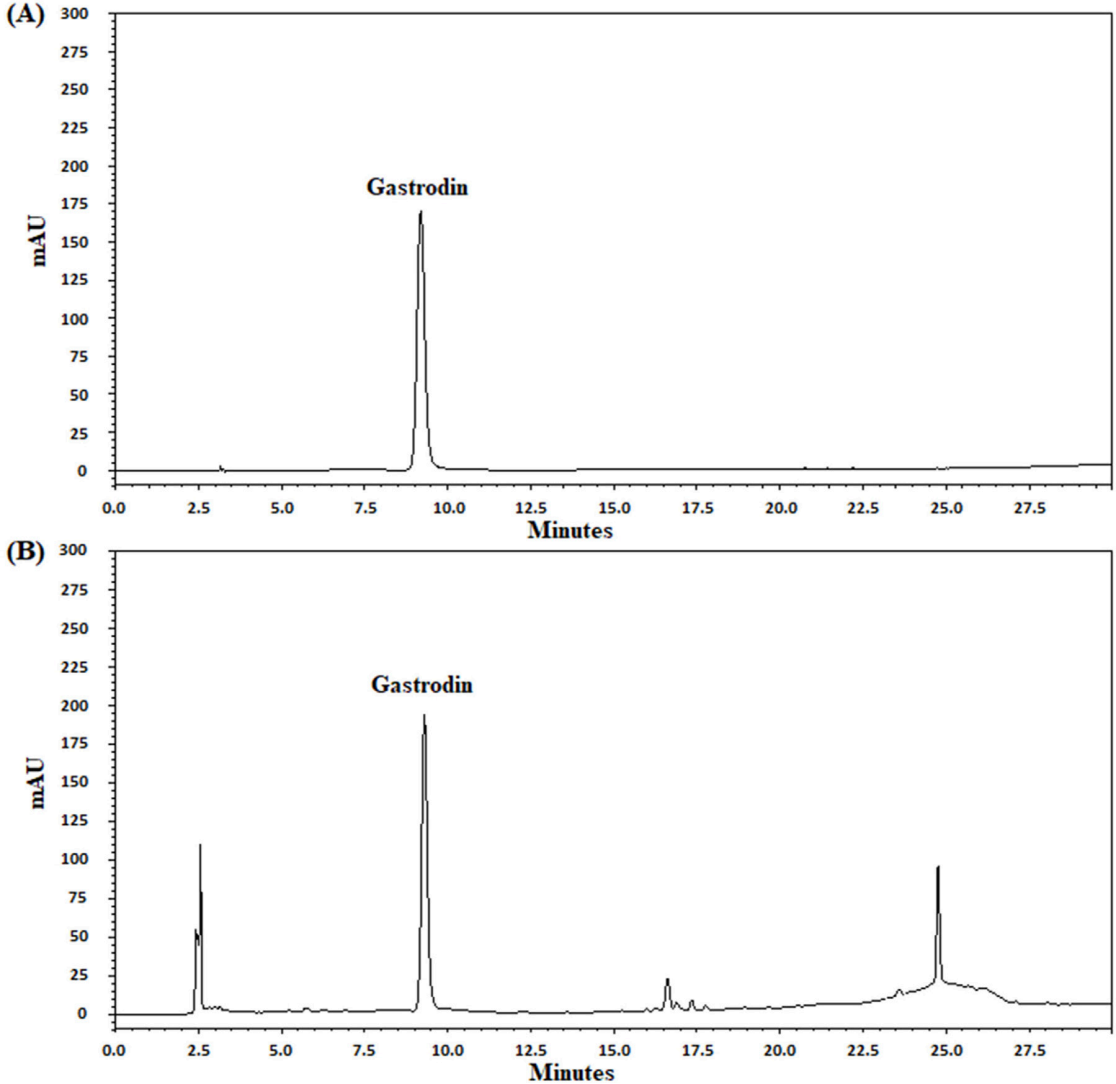
UPLC chromatograms of gastrodin in the TM extract. UPLC chromatograms **(A, B)** indicate the standard (gastrodin) and TM extract solutions, respectively. mAU, milli-absorbance unit.

In UPLC analysis, gastrodin retention times from the standard and TM extract solutions were 9.21 and 9.30 min, respectively. Moreover, the gastrodin content in the TM extract was 0.21 mg/mL (Figures 1A, B).

### Effects of TM treatments on brain infarcts

**FIGURE 2:**
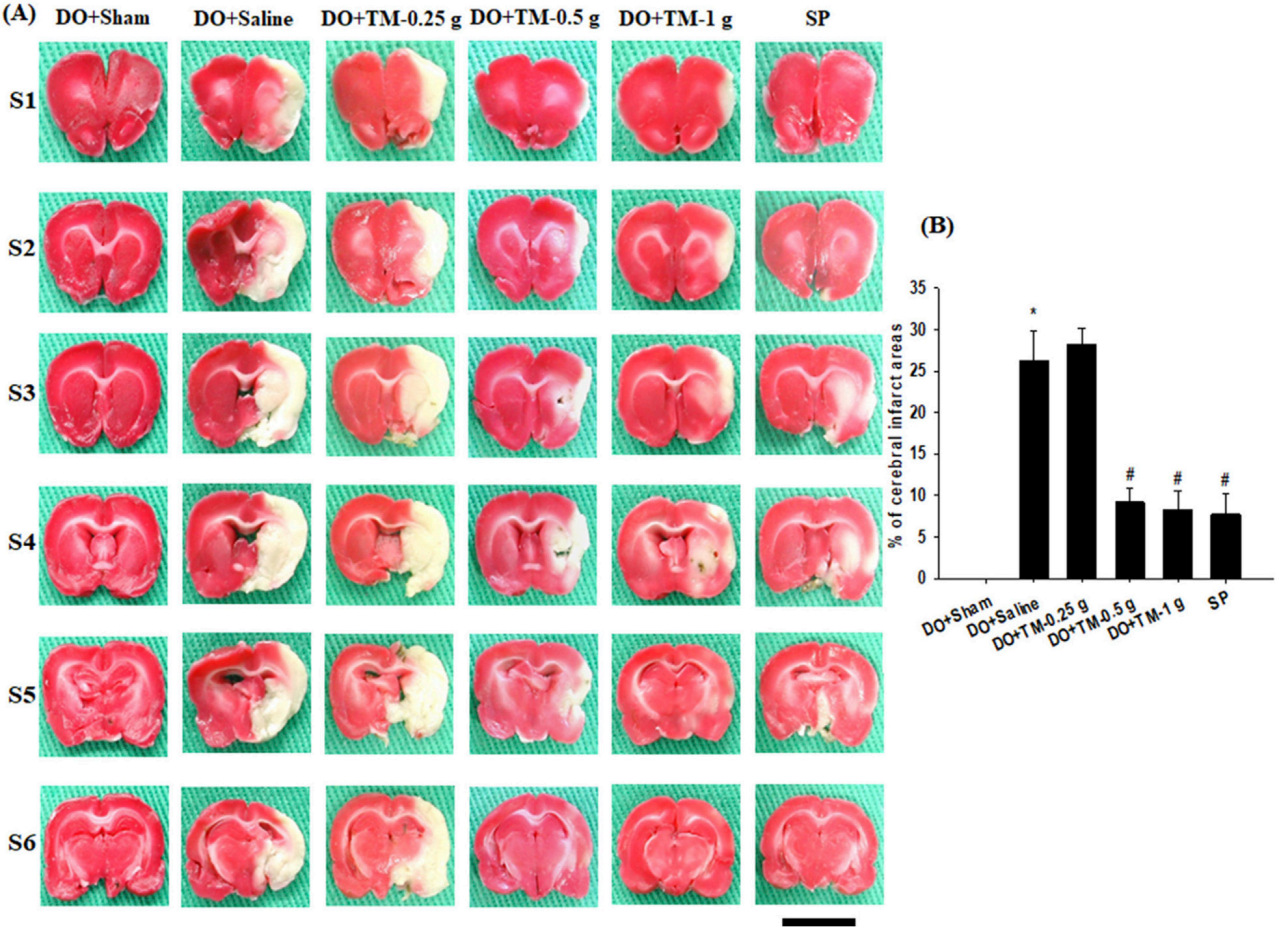
Effects of DO + TM-0.5 g and DO + TM-1 g treatments on cerebral infarct. **(A)** Selected TTC-stained brain slices (S1–S6) of the DO + Sham, DO + Saline, DO + TM-0.25 g, DO + TM-0.5 g, DO + TM-1 g, and SP groups (n = 6) reveal infarct areas (pale white) and normal tissues (dark red). **(B)** The bar graph shows the infarct percentages in the experimental groups 2 days after reperfusion. *P < 0.05 vs. DO + Sham; ^#^ *P* < 0.05 vs. DO + Saline. Scale bar indicates 1 cm.

TTC staining of coronal sections after 2 days of reperfusion revealed no infarct area in the DO + Sham group. By contrast, the infarct areas of the brain coronal sections were significantly higher in the DO + Saline group than in the DO + Sham group (*P* < 0.05) and were significantly lower in the DO + TM-0.5 g, DO + TM-1 g, and SP groups than in the DO + Saline group (all *P* < 0.05; Figures 2A, B).

### Effects of TM treatments on neurological deficits

The NFSs were markedly greater in the DO + Saline group compared to those in the DO + Sham group (*P* < 0.05). In addition, compared with the DO + Saline group, the NFSs were significantly lower in the DO + TM-0.5 g, DO + TM-1 g, and SP groups (all *P* < 0.05; Table 2) at 1 and 2 days after ischemic stroke.

**TABLE 2:** The NFSs in the experimental groups (n = 16).

Group	DO + Sham	DO + Saline	DO + TM-025 g	DO + TM-0.5 g	DO + TM-1 g	SP
Day 1	0.0 (0–0)	8.0 (7–9)*	8.0 (7–10)	4.5 (4–6)^#^	5.0 (3–6)^#^	5.0 (3–6)^#^
Day 2	0.0 (0–0)	8.0 (7–11)*	8.0 (7–11)	4.0 (3–6)^#^	4.0 (2–6)^#^	4.0 (3–5)^#^

Each value was expressed as median (range). NFSs, neurological function scores; *P < 0.05 vs. DO + Sham; ^#^ *P* < 0.05 vs. DO + Saline.

### Effects of TM treatments on the levels of MAPKs, TRAF6, TRAF3, and T3JAM

**FIGURE 3:**
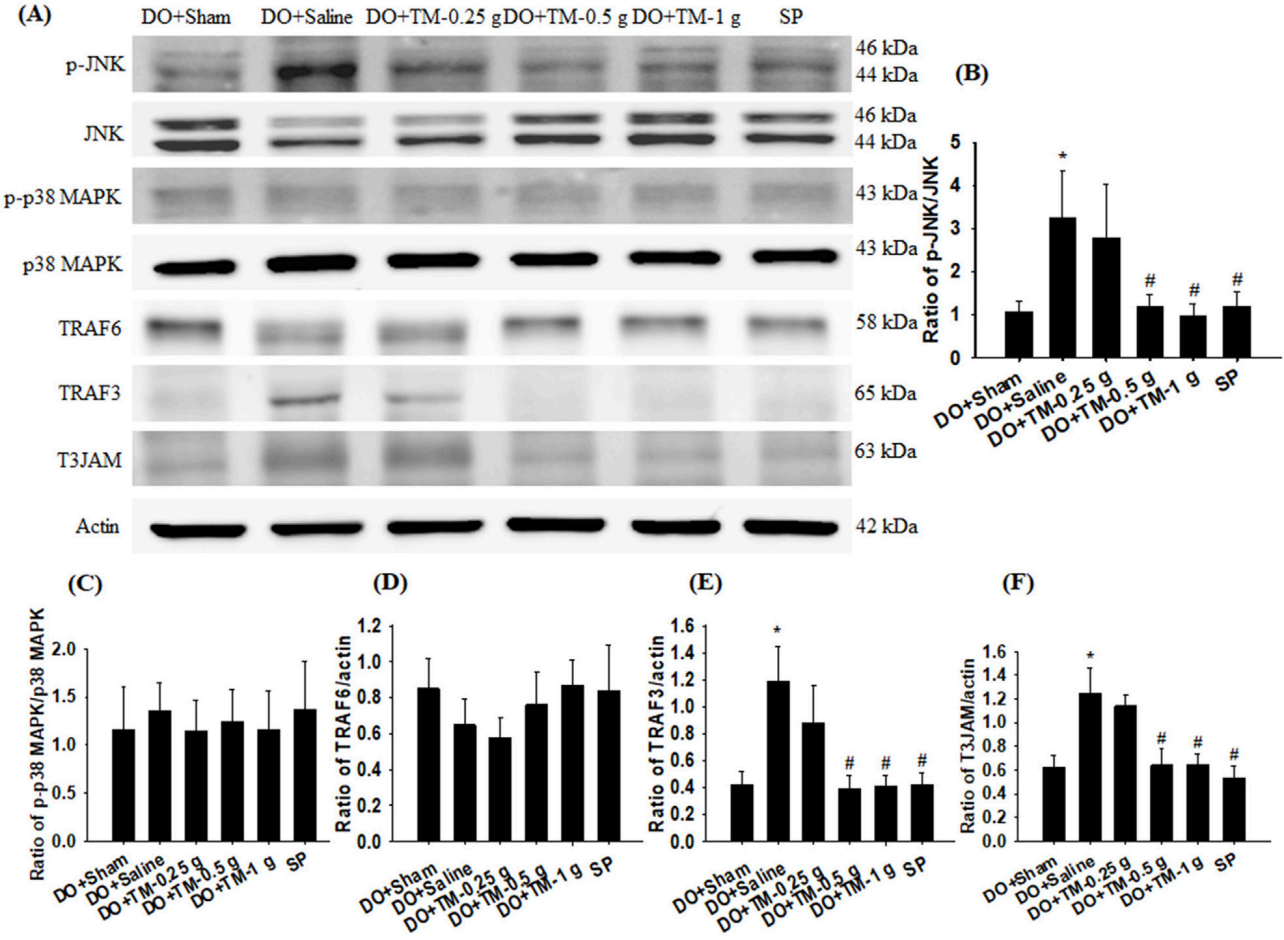
Protein expression of MAPKs, TRAF6, TRAF3, and T3JAM in the cortical ischemic rim. **(A)** Selected Western blot images reveal p-JNK, JNK, p-p38 MAPK, p38 MAPK, TRAF6, TRAF3, T3JAM, and actin expression in the cortical ischemic rim in the DO + Sham, DO + Saline, DO + TM-0.25 g, DO + TM-0.5 g, DO + TM-1 g, and SP groups (n = 5) 2 days after reperfusion. Quantification of the ratios of **(B)** p-JNK to JNK, **(C)** p-p38 MAPK to p38 MAPK, **(D)** TRAF6 to actin, **(E)** TRAF3 to actin, and **(F)** T3JAM to actin was performed in the experimental groups. *P < 0.05 vs. DO + Sham; ^#^ *P* < 0.05 vs. DO + Saline.

The ratios of p-JNK to JNK, TRAF3 to actin, and T3JAM to actin in the cortical ischemic rim were significantly higher in the DO + Saline group than in the DO + Sham group (all *P* < 0.05). In addition, there were significantly lower in the ratios of the aforementioned proteins in the DO + TM-0.5 g, DO + TM-1 g, and SP groups than in the DO + Saline group 2 days after reperfusion (all *P* < 0.05; Figures 3A, B, E, F and Table 3). The ratios of p-p38 MAPK to p38 MAPK and TRAF6 to actin in the cortical ischemic rim were not significantly different in the experimental groups (*P* > 0.05; Figures 3A, C, D and Table 3).

**TABLE 3:** The ratios of target proteins in Western blot analysis in the experimental groups (n = 5).

Group	DO + Sham	DO + Saline	DO + TM-0.25 g	DO + TM-0.5 g	DO + TM-1 g	SP
p-JNK/JNK	1.07 ± 0.23	3.24 ± 1.08*	2.79 ± 1.23	1.19 ± 0.28^#^	0.97 ± 0.28^#^	1.19 ± 0.34^#^
p-p38/p38	1.16 ± 0.44	1.35 ± 0.30	1.14 ± 0.33	1.24 ± 0.34	1.16 ± 0.40	1.37 ± 0.50
TRAF6/actin	0.85 ± 0.17	0.65 ± 0.14	0.58 ± 0.11	0.76 ± 0.18	0.87 ± 0.14	0.84 ± 0.25
TRAF3/actin	0.42 ± 0.10	1.19 ± 0.26*	0.88 ± 0.28	0.39 ± 0.10^#^	0.41 ± 0.08^#^	0.42 ± 0.09^#^
T3JAM/actin	0.62 ± 0.10	1.25 ± 0.21*	1.14 ± 0.10	0.64 ± 0.14^#^	0.65 ± 0.09^#^	0.53 ± 0.10^#^
p-NF-κB/NF-κB	0.47 ± 0.05	1.77 ± 0.48*	1.52 ± 0.23	0.47 ± 0.07^#^	0.42 ± 0.05^#^	0.49 ± 0.06^#^
NLRP3/actin	0.65 ± 0.05	1.49 ± 0.21*	1.51 ± 0.24	0.64 ± 0.05^#^	0.70 ± 0.12^#^	0.63 ± 0.08^#^
Iba1/actin	0.79 ± 0.09	1.27 ± 0.16*	1.25 ± 0.10	0.80 ± 0.06^#^	0.72 ± 0.13^#^	0.75 ± 0.12^#^
VEGF-A/actin	1.42 ± 0.19	0.55 ± 0.18*	0.47 ± 0.11	0.95 ± 0.20^#^	1.35 ± 0.14^#^	1.02 ± 0.21^#^
CD86/actin	0.52 ± 0.03	1.08 ± 0.19*	1.01 ± 0.09	0.58 ± 0.07^#^	0.57 ± 0.09^#^	0.55 ± 0.06^#^
TNF-α/actin	0.37 ± 0.05	1.07 ± 0.04*	0.97 ± 0.12	0.40 ± 0.07^#^	0.41 ± 0.05^#^	0.42 ± 0.09^#^
IL-1β/actin	0.40 ± 0.03	0.72 ± 0.11*	0.72 ± 0.09	0.43 ± 0.04^#^	0.43 ± 0.05^#^	0.37 ± 0.06^#^
IL-6/actin	0.33 ± 0.07	0.67 ± 0.09*	0.65 ± 0.06	0.33 ± 0.04^#^	0.34 ± 0.05^#^	0.31 ± 0.03^#^
YM-1/2/actin	0.93 ± 0.14	0.29 ± 0.12*	0.28 ± 0.12	0.73 ± 0.09^#^	1.06 ± 0.28^#^	0.83 ± 0.17^#^
IL-10/actin	0.62 ± 0.04	0.46 ± 0.06*	0.53 ± 0.06	0.65 ± 0.04^#^	0.68 ± 0.05^#^	0.67 ± 0.09^#^
NGF/actin	0.82 ± 0.07	0.41 ± 0.03*	0.42 ± 0.06	0.74 ± 0.10^#^	0.92 ± 0.12^#^	0.74 ± 0.15^#^

Each value was expressed as mean ± standard deviation. p-p38/p38, p-p38 MAPK/p38 MAPK; p-NF-κB/NF-κB, p-NF-κB p65/NF-κB p65. *P < 0.05 vs. DO + Sham; ^#^ *P* < 0.05 vs. DO + Saline.

### Effects of TM treatments on the levels of p-NF-κB p65, NF-κB p65, NLRP3, ionized calcium-binding adapter molecule 1, and vascular endothelial growth factor A

**FIGURE 4:**
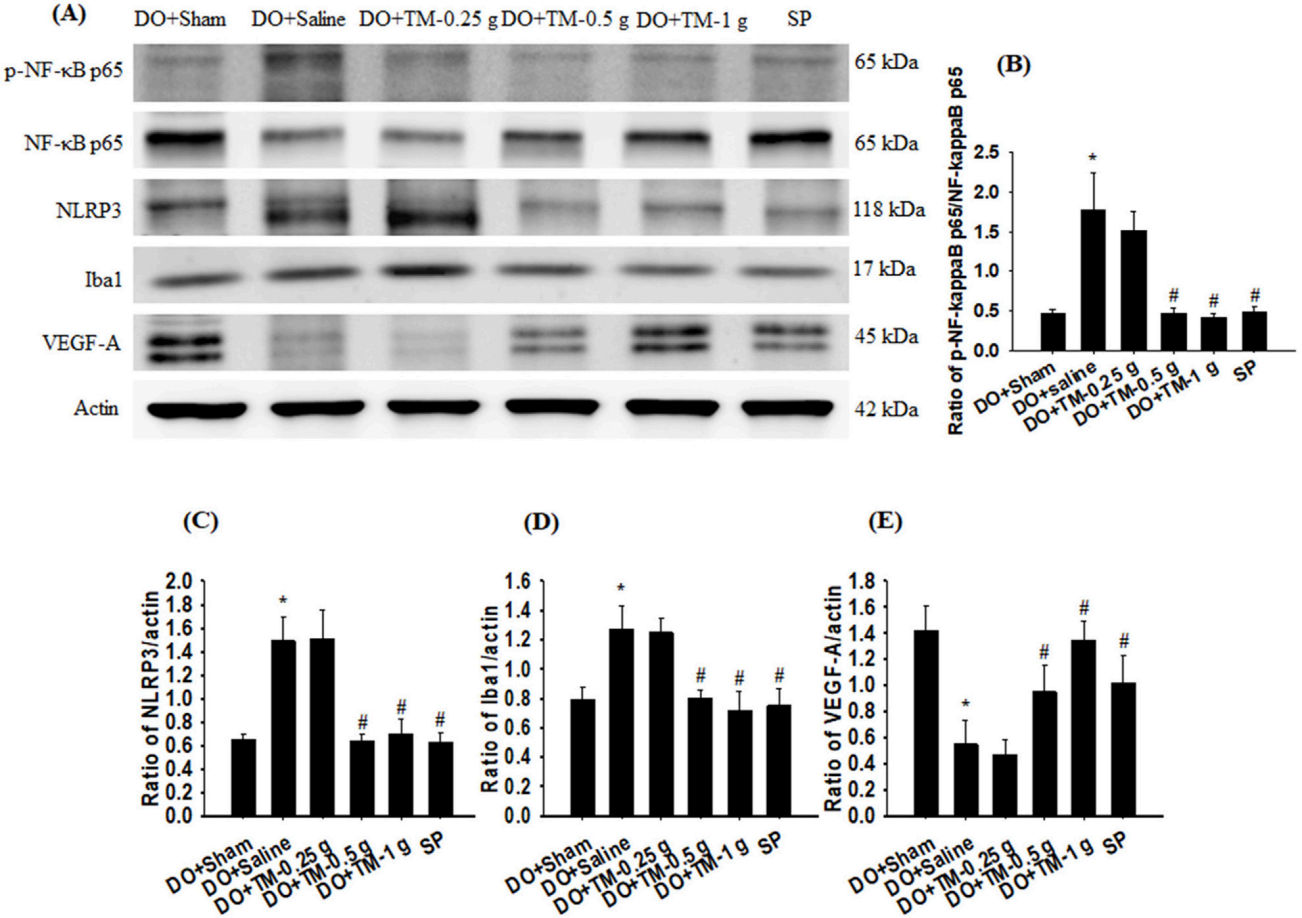
Protein expression of p-NF-κB p65, NF-κB p65, NLRP3, Iba1, Nrf2, and VEGF-A in the cortical ischemic rim. **(A)** Selected Western blot images reveal p-NF-κB p65, NF-κB p65, NLRP3, Iba1, Nrf2, VEGF-A, and actin expression in the cortical ischemic rim in the DO + Sham, DO + Saline, DO + TM-0.25 g, DO + TM-0.5 g, DO + TM-1 g, and SP groups (n = 5) 2 days after reperfusion. Quantification of the ratios of **(B)** p-NF-κB p65 to NF-κB p65, **(C)** NLRP3 to actin, **(D)** Iba1 to actin, and **(E)** VEGF-A to actin was performed in the experimental groups. *P < 0.05 vs. DO + Sham; ^#^ *P* < 0.05 vs. DO + Saline.

The ratios of p-NF-κB p65 to NF-κB p65, NLRP3 to actin, and ionized calcium-binding adapter molecule 1 (Iba1) to actin in the cortical ischemic rim were significantly higher in the DO + Saline group than in the DO + Sham group (all *P* < 0.05). In addition, the ratios of the aforementioned proteins were significantly lower in the DO + TM-0.5 g, DO + TM-1 g, and SP groups compared to those in the DO + Saline group 2 days after reperfusion (all *P* < 0.05; Figures 4A–D and Table 3). By contrast, the ratio of vascular endothelial growth factor A (VEGF-A) to actin in the cortical ischemic rim was significantly lower in the DO + Saline group than in the DO + Sham group (*P* < 0.05) and was significantly higher in the DO + TM-0.5 g, DO + TM-1 g, and SP groups than in the DO + Saline group (*P* < 0.05; Figures 4A, E and Table 3).

### Effects of TM treatments on the levels of CD86, TNF-α, IL-1β, IL-6, YM-1/2, IL-10, and nerve growth factor

**FIGURE 5:**
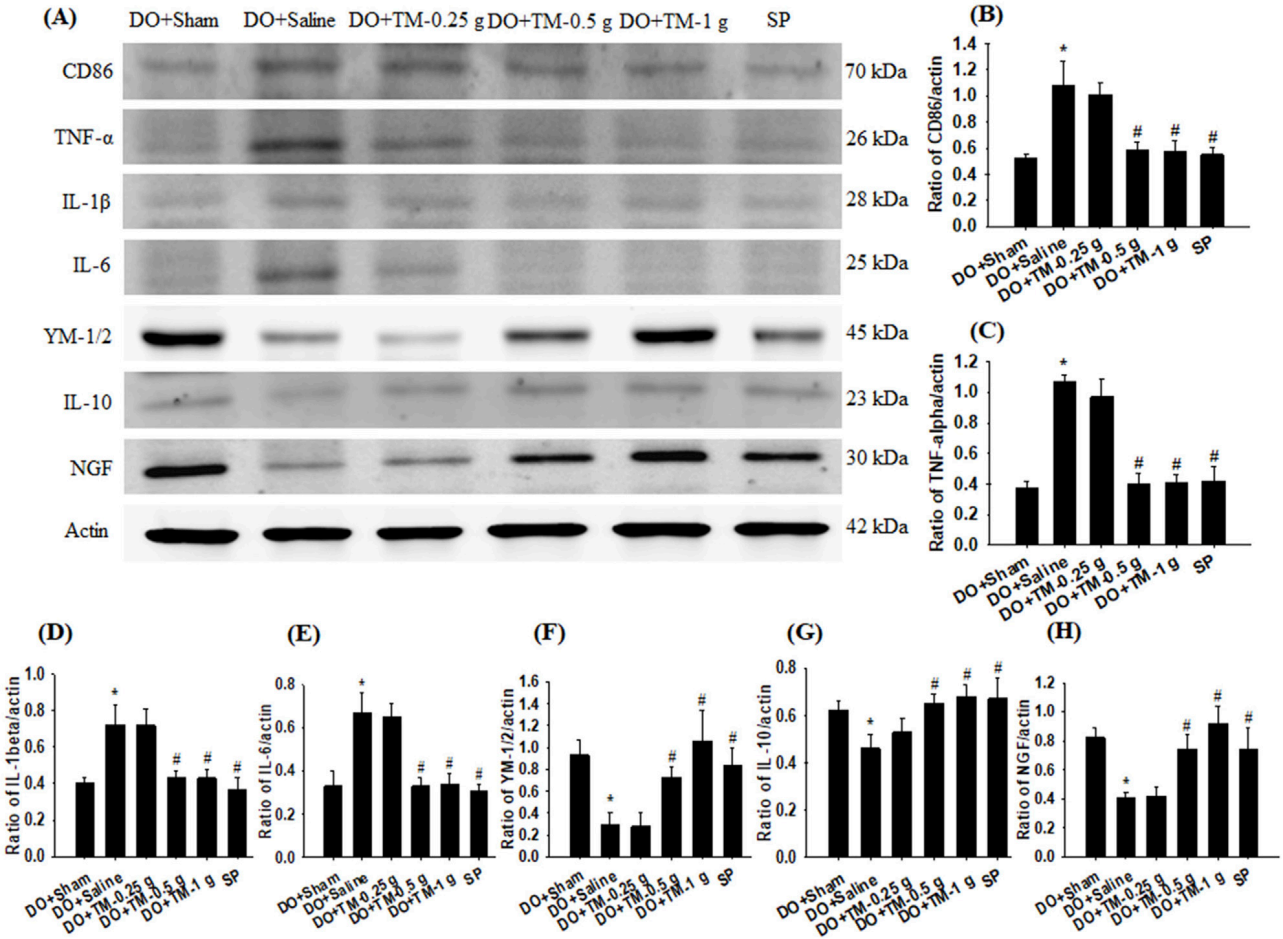
Protein expression of CD86, TNF-α, IL-1β, IL-6, YM-1/2, IL-10, and NGF in the cortical ischemic rim. **(A)** Selected Western blot images reveal CD86, TNF-α, IL-1β, IL-6, YM-1/2, IL-10, NGF, and actin expression in the cortical ischemic rim in the DO + Sham, DO + Saline, DO + TM-0.25 g, DO + TM-0.5 g, DO + TM-1 g, and SP groups (n = 5) 2 days after reperfusion. Quantification of the ratios of **(B)** CD86 to actin, **(C)** TNF-α to actin, **(D)** IL-1β to actin, **(E)** IL-6 to actin, **(F)** YM-1/2 to actin, **(G)** IL-10 to actin, and **(H)** NGF to actin was performed in the experimental groups. *P < 0.05 vs. DO + Sham; ^#^ *P* < 0.05 vs. DO + Saline.

The ratios of CD86 to actin, TNF-α to actin, IL-1β to actin, and IL-6 to actin in the cortical ischemic rim were significantly higher in the DO + Saline group than in the DO + Sham group (all P < 0.05) and were significantly lower in the DO + TM-0.5 g, DO + TM-1 g, and SP groups than in the DO + Saline group 2 days after reperfusion (all *P* < 0.05; Figures 5A–E and Table 3). By contrast, the ratios of YM-1/2 to actin, IL-10 to actin, and nerve growth factor (NGF) to actin in the cortical ischemic rim were significantly lower in the DO + Saline group than in the DO + Sham group (all *P* < 0.05) and were significantly higher in the DO + TM-0.5 g, DO + TM-1 g, and SP groups than in the DO + Saline group (all *P* < 0.05; Figures 5A, F–H and Table 3).

### Effects of TM treatments on the levels of TLR4/Iba1-, CD86/Iba1-, p-NF-κB p65/Iba1-, YM-1/2/Iba1-, and IL-10/neuronal nuclei-positive cells

**FIGURE 6:**
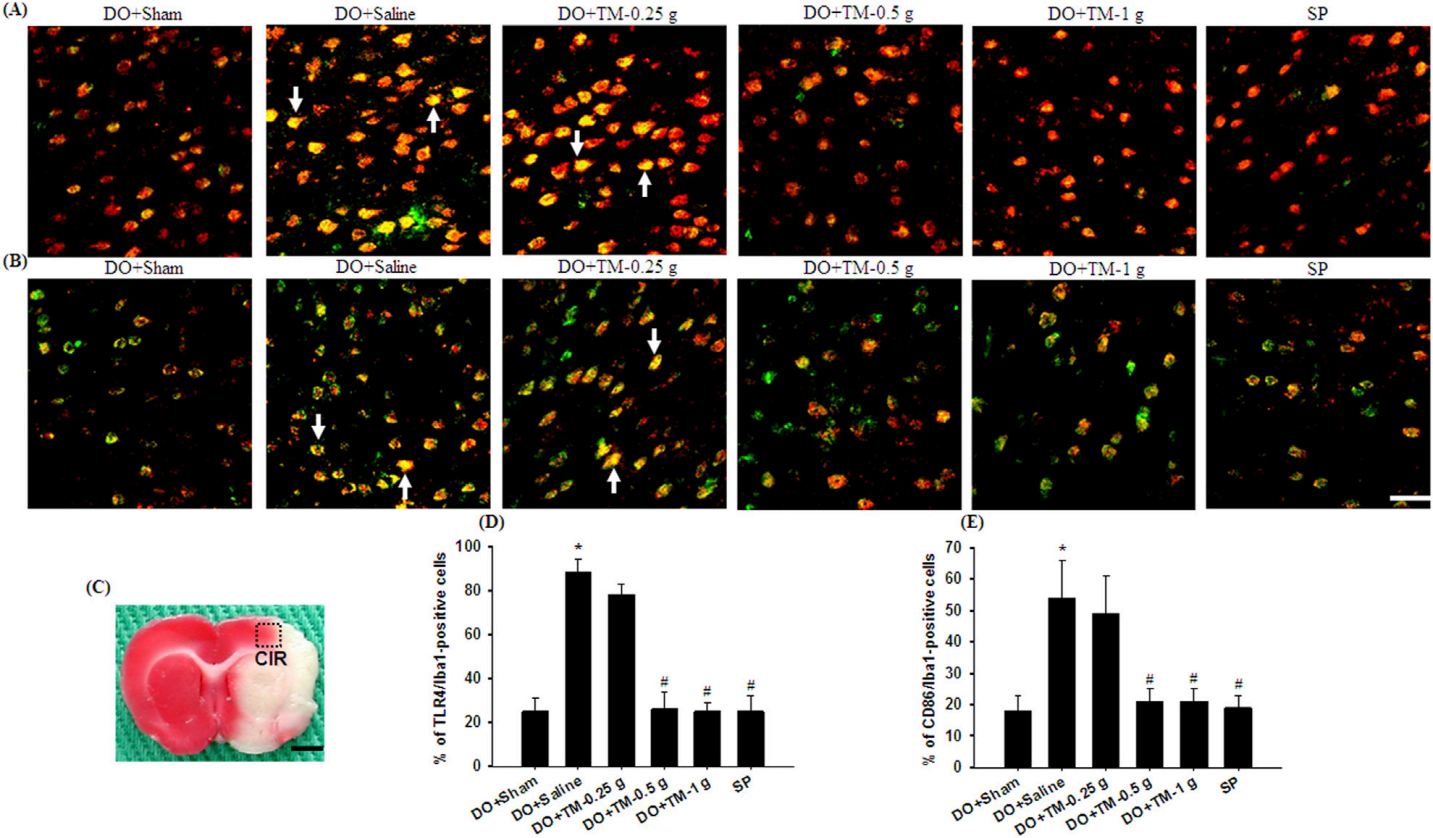
The percentages of TLR4/Iba1-and CD86/Iba1-positive cells in the cortical ischemic rim. Representative IF images reveal **(A)** TLR4 (red)/Iba1 (green) and **(B)** CD86 (red)/Iba1 (green) expression in the cortical ischemic rim in the DO + Sham, DO + Saline, DO + TM-0.25 g, DO + TM-0.5 g, DO + TM-1 g, and SP groups (n = 5) 2 days after reperfusion. Arrows in **(A, B)** point to TLR4/Iba1 (yellow)- and CD86/Iba1 (yellow)-positive cells, respectively. **(C)** The dashed line square in a representative coronal section shows the region where immunopositive cells were detected. CIR, cortical ischemic rim. Dashed line square equals 1 mm^2^. Scale bar indicates **(B)** 50 μm and **(C)** 2 mm. Quantification of the percentages of **(D)** TLR4/Iba1-and **(E)** CD86/Iba1-positive cells was performed in the experimental groups. *P < 0.05 vs. DO + Sham; ^#^ *P* < 0.05 vs. DO + Saline.

**FIGURE 7:**
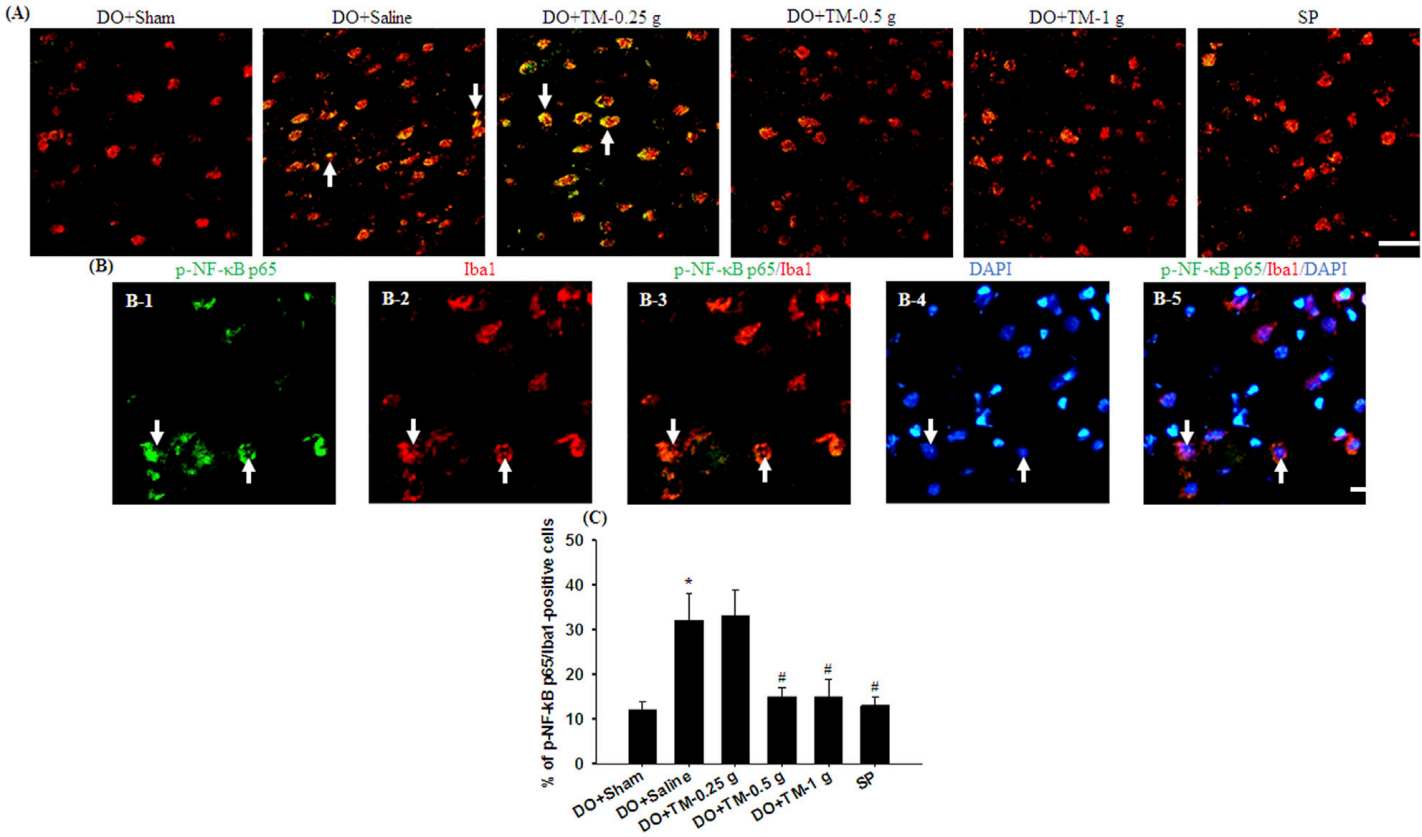
The percentage of p-NF-κB p65/Iba1-positive cells in the cortical ischemic rim. **(A)** Representative IF images reveal p-NF-κB p65 (green)/Iba1 (red) expression in the cortical ischemic rim in the DO + Sham, DO + Saline, DO + TM-0.25 g, DO + TM-0.5 g, DO + TM-1 g, and SP groups (n = 5) 2 days after reperfusion. Arrows in **(A)**, **(B-1)**, **(B-2)**, **(B-3)**, **(B-4)**, and **(B-5)** point to p-NF-κB p65/Iba1 (yellow)-, p-NF-κB p65 (green)-, Iba1 (red)-, p-NF-κB p65/Iba1 (yellow)-, DAPI (blue)-, and p-NF-κB p65/Iba1/DAPI (light pink)-positive cells, respectively. Scale bar indicates **(A)** 50 μm and **(B)** 10 μm. Quantification of the percentage of **(C)** p-NF-κB p65/Iba1-positive cells was performed in the experimental groups. DAPI, 4′,6-diamidino-2-phenylindole (used as a nuclear counterstain). *P < 0.05 vs. DO + Sham; ^#^ *P* < 0.05 vs. DO + Saline.

**FIGURE 8:**
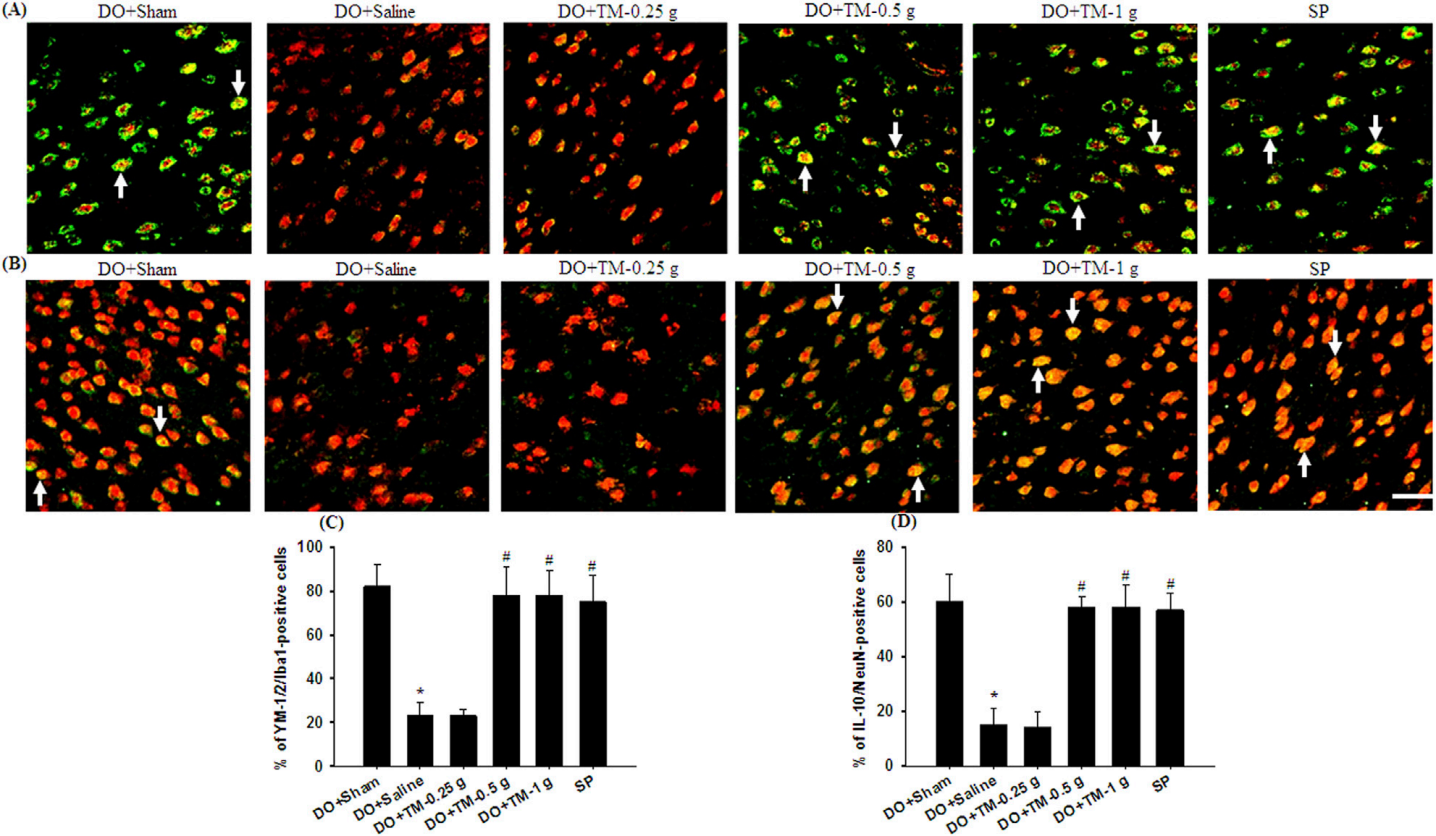
The percentages of YM-1/2/Iba1-and IL-10/NeuN-positive cells in the cortical ischemic rim. Representative IF images reveal **(A)** YM-1/2 (green)/Iba1 (red) and **(B)** IL-10 (green)/NeuN (red) expression in the cortical ischemic rim in the DO + Sham, DO + Saline, DO + TM-0.25 g, DO + TM-0.5 g, DO + TM-1 g, and SP groups (n = 5) 2 days after reperfusion. Arrows in **(A)** and **(B)** point to YM-1/2/Iba1 (yellow)- and IL-10/NeuN (yellow)-positive cells, respectively. Scale bar indicates 50 μm. Quantification of the percentages of **(C)** YM-1/2/Iba1-and **(D)** IL-10/NeuN-positive cells was performed in the experimental groups. *P < 0.05 vs. DO + Sham; ^#^ *P* < 0.05 vs. DO + Saline.

TLR4-, CD86^−^, p-NF-κB p65-, and partial YM-1/2-positive cells were colocalized with Iba1 in the cortical ischemic rim (Figures 6A, B, 7A, B-1–B-3, 8A). In addition, p-NF-κB p65/Iba1 double-labeled cells were examined in the nucleus (Figures 7B-4, B-5) and IL-10-positive cells were colocalized with neuronal nuclei (NeuN) in the cortical ischemic rim (Figure 8B). All the above-mentioned double-labeled cells were examined in the selected cortical ischemic rim (Figure 6C). The percentages of TLR4/Iba1-, CD86/Iba1-, and p-NF-κB p65/Iba1-positive cells in the cortical ischemic rim were significantly greater in the DO + Saline group compared to those in the DO + Sham group. In addition, the percentages of above-mentioned positive cells were significantly lower in the DO + TM-0.5 g, DO + TM-1 g, and SP groups than in the DO + Saline group 2 days after reperfusion (all *P* < 0.05; Figures 6A, B, D, E, 7A, C). By contrast, the percentages of YM-1/2/Iba1-and IL-10/NeuN-positive cells in the cortical ischemic rim were significantly lower in the DO + Saline group compared to those in the DO + Sham group and were significantly greater in the DO + TM-0.5 g, DO + TM-1 g, and SP groups compared to those in the DO + Saline group (all *P* < 0.05; Figures 8A–D).

## Discussion

Post-ischemic inflammation plays a vital role in cerebral infarction in the acute stage of transient cerebral ischemia (Zhang et al., 2021). Furthermore, the interaction between TLR4-and JNK (p38 MAPK)-mediated inflammatory signaling pathways induces the downstream activation of the NF-κB pathway, amplifying microglial activation and exacerbating I/R injury in acute ischemic stroke (Cheng et al., 2021; Zhang et al., 2021). UPLC analysis revealed that gastrodin (a marker of the TM extract) contents were 0.42, 0.84, and 1.68 mg in 0.25, 0.5, and 1 g/kg of the TM extract solutions, respectively. Moreover, our results revealed that cerebral infarction and neurological deficits were predominantly created 2 days after transient MCA occlusion. However, 0.5 and 1 g/kg, but not 0.25 g/kg, of the TM extract significantly reduced infarct areas and promoted neurological function recovery. Moreover, Western blotting and double IF staining revealed that the levels of p-JNK/JNK and TLR4/Iba1 (microglia marker), but not of p-p38 MAPK/p38 MAPK, were markedly increased in the cortical ischemic rim. However, 0.5 and 1 g/kg TM restored these levels of p-JNK and TLR4 in the cortical ischemic rim, reducing the interaction between JNK- and TLR4-mediated inflammatory cascades in microglia in the acute stage after transient cerebral ischemia. Overall, the findings imply that TM treatments protect against cerebral infarction at least partly by suppressing the interaction between JNK and TLR4 in microglia in the cortical ischemic rim 2 days after transient MCA occlusion.

TLR4 expressed on activated microglia recognizes DAMPs and then triggers MyD88/TRAF6- and TRAF3/T3JAM-mediated inflammatory signaling pathways (Wang et al., 2011; Cheng et al., 2021). TLR4 expression plays a vital role in innate immunity. Moreover, TLR4-induced neuroinflammation through downstream NF-κB signaling activation is positively correlated with ischemic stroke severity (Wang et al., 2015). The results of the current study indicated that the expression of TRAF3, but not of TRAF6, was markedly upregulated in the cortical ischemic rim, whereas TM treatments reversed the increased expression of TRAF3 and T3JAM in the acute stage of transient MCA occlusion. TRAF3 is a central regulator of ischemic signaling pathways, and it participates in apoptosis, inflammation, and oxidative stress events in acute ischemic stroke (Gong et al., 2015). T3JAM, a coiled-coil membrane protein, is predominantly expressed in the immune system and interacts with TRAF3 (Li et al., 2019). Their interaction in the cytosol amplifies JNK activation (Dadgostar et al., 2003) and causes TLR4-induced nuclear translocation of the NF-κB p65 subunit in acute cerebral ischemia (Gong et al., 2015; Cheng et al., 2021). The NF-κB family comprises five members: p50, P52, p65, c-Rel, and Rel-B. In cerebral I/R injury, p65 phosphorylation enhances the stability of the NF-κB complex, which is translocated to the nucleus for inducing the expression of genes encoding proinflammatory factors. Thus, p-NF-κB p65 is considered as an indicator of NF-κB activation (Wang et al., 2018). NF-κB p65, an essential transcriptional regulator, binds to IκB in the inactive state under normal conditions, whereas the NF-κB p65/IκB complex is dissociated through IκB degradation following cerebral I/R stimulation; the free NF-κB p65 complex is translocated to the nucleus in the activation (phosphorylation) state (Stephenson et al., 2000; Gong et al., 2023). In addition, p65 phosphorylation is essential for the nuclear translocation of NF-κB p65 and the transcription of its inflammatory mediators, which induces microglial activation and polarization toward the M1 phenotype, aggravating cerebral I/R injury (Yang et al., 2021). NF-κB p65 translocation also promotes polarization toward the M1 phenotype and inhibits conversion into the M2 phenotype in activated microglia (Li et al., 2022). The NLRP3 inflammasome, a critical component in the innate immune system, participates the progression of microglial polarization in the ischemic area in the acute stage of cerebral ischemia (Bian et al., 2021). The NLRP3 inflammasome mainly localized in the microglia is generated by the activation of the NF-κB signaling pathway; it then upregulates IL-1β and IL-18 expression, promoting M1 microglial polarization after MCA occlusion (Bian et al., 2021; Xue et al., 2023). By contrast, the inhibition of NLRP3 inflammasome activity suppresses microglial activation and M1 polarization, thereby downregulating the inflammatory response in the penumbra and reducing the infarct size in the early stages of cerebral ischemia (Xue et al., 2023). Thus, the pharmacological modulation of the NF-κB p65/NLRP3-mediated pathway and subsequent promotion of M1 to M2 polarization can reduce neurological deficits and cerebral infarct and is a promising therapeutic strategy in the acute stage of transient cerebral ischemia (Song et al., 2021; Li et al., 2022). Furthermore, studies have shown that the promotion of the microglial polarization shift from M1 to M2 phenotype in the ischemic area in the acute phase of cerebral ischemia is critical for long-term neurological function recovery (Gelosa et al., 2019; Suofu et al., 2023). In the present study, p-NF-κB p65-positive cells were colocalized with Iba1 in the nuclei, and these p-NF-κB p65/Iba1 double-labeled cells were predominantly detected in the cortical ischemic rim. Western blotting results further revealed markedly increased levels of p-NF-κB p65/NF-κB p65, NLRP3, and Iba1 in the cortical ischemic rim. Elevated Iba1 levels are commonly used as a marker of microglial activation (Jurga et al., 2020). Taken together, these results revealed that p-NF-κB p65 was translocated to the nucleus and induced the transcription of the downstream target NLRP3, thereby upregulating NF-κB p65/NLRP3 signaling in activated microglia. However, TM treatments restored the increased levels of the aforementioned proteins in the cortical ischemic rim 2 days after transient MCA occlusion. According to these findings, we suggest that TM treatments ameliorate cerebral infarction possibly by downregulating JNK/TLR4/T3JAM-mediated signaling in activated microglia. Furthermore, TM treatments exert neuroprotective effects by suppressing M1 microglial polarization at least partly by downregulating JNK/TLR4/T3JAM-mediated NF-κB p65/NLRP3 signaling in the cortical ischemic rim in the acute stage of transient MCA occlusion.

Activated microglia are highly plastic cells and undergo morphological transformation into either the proinflammatory M1 or anti-inflammatory M2 phenotype (Jover-Mengual et al., 2021). Microglia undergo polarization toward the M2 phenotype at 1–3 days after cerebral ischemia but gradually shift to the M1 phenotype 3–14 days after ischemia (Zhao et al., 2019; Ran et al., 2021). M1 microglia (with TNF-α or CD86 as markers) generate pro-inflammatory factors, such as TNF-α, IL-1β, IL-6, and interferon-γ, which promote inflammation and exacerbate I/R injury and cerebral infarction. By contrast, M2 microglia (with CD206 or YM-1/2 as markers) generate anti-inflammatory factors, such as transforming growth factor-β, IL-4, and IL-10, and neurotrophic factors, such as NGF and VEGF, which promote neuronal proliferation, differentiation, and survival and restoration of the blood–brain barrier, thereby promoting tissue recovery (Wijaya et al., 2021; Guo et al., 2022; Li et al., 2022; Wan et al., 2022). In our study, Western blotting and IF revealed that in the cortical ischemic rim, CD86, CD86/Iba1, TNF-α, IL-1β, and IL-6 levels were markedly upregulated, whereas YM-1/2, YM-1/2/Iba1, IL-10, IL-10/NeuN (mature neuron marker), and NGF levels were significantly downregulated. The level of VEGF-A, a member of the VEGF family, also markedly reduced in the acute stage of transient MCA occlusion. However, TM treatments reversed all these changes 2 days after reperfusion. Accordingly, we infer that TM treatments protect against M1 microglia-induced inflammation, possibly by downregulating TNF-α, IL-1β, and IL-6 expression and upregulating IL-10, NGF, and VEGF-A expression in the cortical ischemic rim. Furthermore, the neuroprotective effects of TM treatments on promoting the microglial polarization shift from M1 to M2 may be partly due to the downregulation of JNK/TLR4/T3JAM/NF-κB-mediated signaling in the cortical ischemic rim 2 days after reperfusion.

**FIGURE 9:**
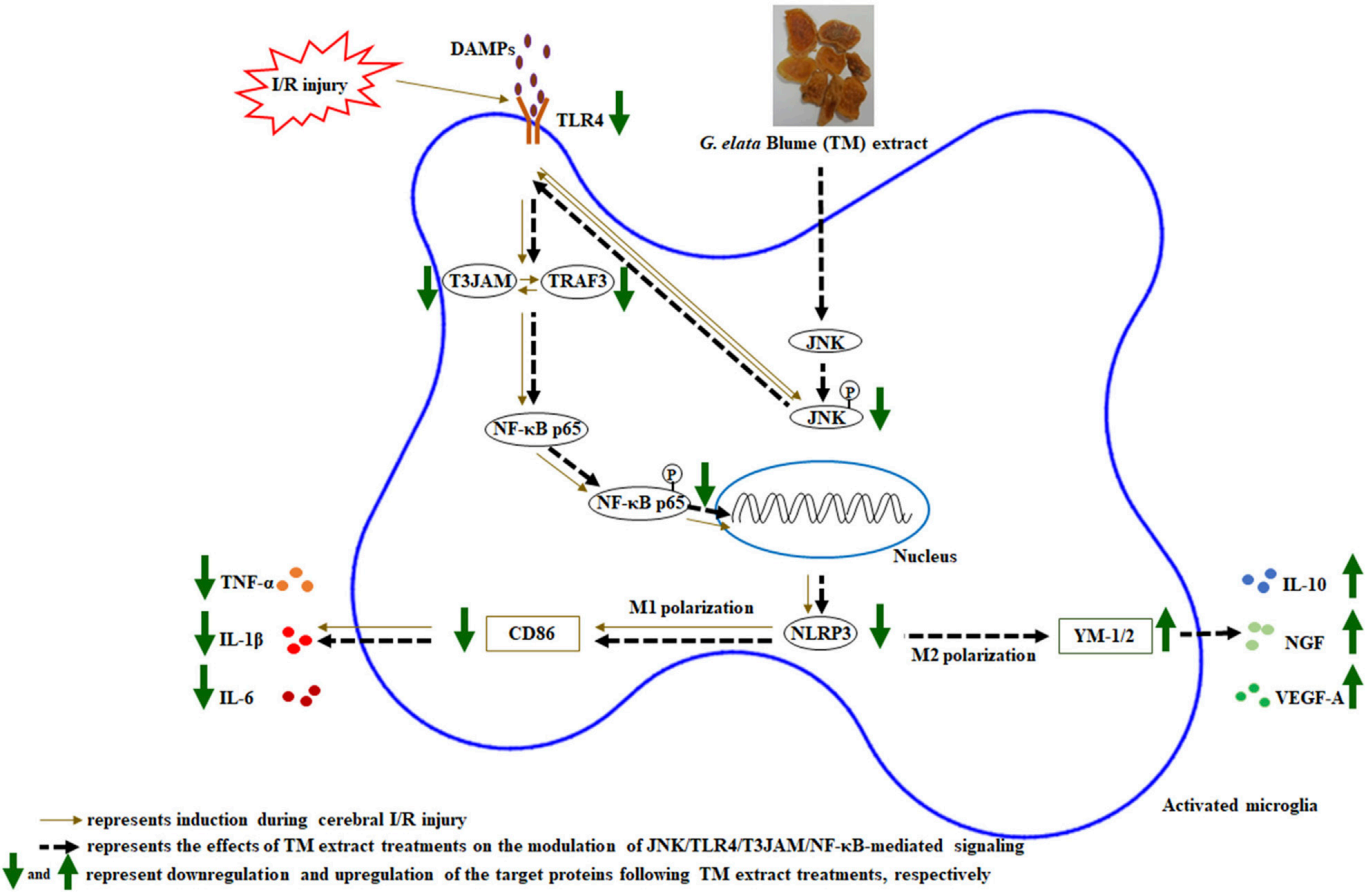
Schematic representation of the possible mechanisms of the TM extract on promoting microglia M2 polarization by inhibiting JNK/TLR4/T3JAM/NF-κB-mediated signaling after transient MCA occlusion. P, phosphorylated.

To identify the detailed reaction mechanisms underlying the anti-infarct effects of TM treatments on the modulation of JNK-related TLR4/T3JAM/NF-κB-mediated signaling 2 days after transient MCA occlusion, we pretreated SP600125 in the SP group. SP600125, a JNK inhibitor, effectively blocks JNK activation in the acute stage of cerebral ischemia (Cheng et al., 2021). TTC staining, Western blotting, and IF assays revealed that the effects of SP treatment on the aforementioned proteins are similar to those of TM treatments and those subsequently reduced cerebral infarct areas and NFSs 2 days after reperfusion. Thus, TM treatments ameliorate cerebral infarction and neurological deficits by inhibiting the JNK signaling pathway in the peri-infarct cortex. Moreover, the neuroprotective effects of TM treatments on promoting microglial M1 to M2 polarization are due to the inhibition of JNK/TLR4/T3JAM/NF-κB-mediated signaling in the cortical ischemic rim 2 days after reperfusion (Figure 9). Previous research showed that TM pretreatments attenuate neurological deficits in the acute phase of transient MCA occlusion (Duan et al., 2015; Seok et al., 2019; Wang et al., 2019) (Table 4). In the present study, TM treatments at the onset of cerebral ischemia can also improve neurological function recovery in the acute phase of transient cerebral ischemia. Furthermore, other studies reported that the bioactive ingredients of the TM extract include gastrodin, 4-hydroxybenzyl alcohol, parishin, and 3,4-dihydroxybenzaldehyde, which exert anti-inflammatory effects by modulating MAPK and NF-κB activation *in vitro* and *in vivo* models (Kim et al., 2012; Xiang et al., 2018; Li et al., 2020; Lin et al., 2021; Ma et al., 2024). In addition, the TM extract and its bioactive ingredients exert anti-inflammatory effects by downregulating a variety of proinflammatory mediators, such as TNF-α, IL-1β, and nitric oxide, in various models of ischemia-induced injury (Li et al., 2018; Seok et al., 2019; Wang et al., 2019; Kim et al., 2020). Thus, we reasonably assume that gastrodin (0.84–1.68 mg/kg) in the TM extract possibly interacts with other bioactive ingredients and subsequently provides neuroprotective effects through modulation of JNK-mediated NF-κB signaling during cerebral ischemic injury. However, further investigation is warranted. In the present study, we further provided evidence that the TM extract protects against cerebral I/R-induced inflammatory responses by inhibiting the JNK-related TLR4/T3JAM/NF-κB signaling pathway. To the best of our knowledge, this is the first study to show that the TM extract promotes microglial M2 polarization by inhibiting JNK-mediated TLR4/T3JAM/NF-κB signaling in acute ischemic stroke.

**TABLE 4:** Effects of the TM extract on neurological deficits in MCA occlusion models.

MCA occlusion models	Treatments	Neurological function tests	NFSs	References
2 h of ischemia followed by 24 h of reperfusion	Pretreatment with the TM extract at 11.4 and 102.6 mg/kg	Bederson’s improving method including forelimb flexion, floor walking, response to vibrissae touch, and body rotation tests	NFSs ↓	(Duan et al., 2015)
2 h of ischemia followed by 24 h of reperfusion	Pretreatment with the TM extract at 200 mg/kg	Longa’s scoring	NFSs ↓	(Wang et al., 2019)
1 h of ischemia followed by 24 h of reperfusion	Pretreatment with the TM extract at 10 g/kg	Corner test Adhesive removal test	NFSs ↓	(Seok et al., 2019)

In conclusion, our findings revealed that the 0.5 and 1 g/kg TM extract reduced the cerebral infarct area and attenuated neurological dysfunction in the acute stage of MCA occlusion. Furthermore, TM treatments protected against cerebral infarction by inhibiting the JNK signaling pathway in the cortical ischemic rim. The neuroprotective effects of TM treatments on promoting M2 microglial polarization are attributed to the inhibition of the JNK/TLR4/T3JAM/NF-κB signaling pathway in the cortical ischemic rim 2 days after reperfusion. Thus, these findings suggest that *G*. *elata* Blume extract treatment is a potential therapeutic strategy for reducing cerebral infarction by downregulating the inflammatory response in the acute stage of cerebral ischemia. However, there are still some limitations in this study. First, we should do more exploration to find the better time point of TM administration following cerebral I/R injury. Second, we mainly focused on the anti-inflammatory effects of TM treatments on inhibition of JNK-mediated TLR4/T3JAM/ NF-κB signaling in acute ischemic stroke, but we could not exclude the possibility of the interaction with other signaling pathways, such as chemokine-mediated signaling. Third, the potential effects of the TM extract on MCA occlusion in rodents may not adequately simulate clinical stroke, which is influenced by a variety of factors, including age, gender, and body conditions. Fourth, the observation time point was short only 48 h and the findings of the present study could not reflect the long-term effects of the TM extract during cerebral ischemia. In addition, Paolicelli et al. reported that microglia are often coexpressing M1 and M2 markers and exist in dynamic and multidimensional states depending on environmental stimuli. Thus, reactive microglia should be focused on the specialized functions instead of simply dividing into the dichotomous M1/M2 polarization paradigm (Paolicelli et al., 2022). Thus, future studies should identify the effects and detailed reaction mechanisms of *G*. *elata* Blume extract on microglial functions in the subacute stage of transient cerebral ischemia.

## Data Availability

The original contributions presented in the study are included in the article/Supplementary Material, further inquiries can be directed to the corresponding author.
