# Supplementary material for: Neuroprotective effects of Gastrodia elata Blume on promoting M2 microglial polarization by inhibiting JNK/TLR4/T3JAM/NF-κB signaling after transient ischemic stroke in rats
Source: Front Pharmacol. 2024 Sep 25;15:1469602. doi: 10.3389/fphar.2024.1469602 (PMC11465390; doi:10.3389/fphar.2024.1469602)
Supplement: Supplementary file 1 [file Table1.DOC]

Supplementary materials

The percentage of cerebral infarct areas

| Group | DO+Sham | DO+Saline | DO+TM-0.25 g | DO+TM-0.5 g | DO+TM-1 g | SP |
| --- | --- | --- | --- | --- | --- | --- |
| 1 | 0 | 30.1 | 27.9 | 8.5 | 7.1 | 9.1 |
| 2 | 0 | 29.9 | 28.2 | 6.5 | 8.5 | 6.0 |
| 3 | 0 | 22.7 | 24.6 | 11.0 | 5.8 | 4.3 |
| 4 | 0 | 24.0 | 28.9 | 8.5 | 9.7 | 5.9 |
| 5 | 0 | 28.0 | 29.0 | 10.6 | 7.1 | 10.4 |
| 6 | 0 | 22.3 | 30.6 | 10.2 | 11.8 | 10.5 |
| **Mean** | **0** | **26.2** | **28.2** | **9.2** | **8.3** | **7.7** |
| **SD** | **0** | **3.6** | **2.0** | **1.7** | **2.2** | **2.6** |
